# Nervous Disorders in General Practice

**Published:** 1934

**Authors:** Frank Bodman

**Affiliations:** Physician, Bristol Homæopathic Hospital


### NERVOUS DISORDERS IN GENERAL PRACTICE.

BY

FRANK BODMAN, M.B., Ch.B., M.R.C.S., L.R.C.P.,

*Physician, Bristol Homœopathic Hospital.*

---

THERE is no question that the common nervous disorders make up a large fraction of our daily work. At a conservative estimate 25 per cent. of the patients encountered in general practice, in my experience, are suffering from functional disorders. No doubt a really psychologically-minded physician would double my figures, but for practical purposes I may say that one in every four patients coming to me for advice requires a longer or shorter explanation and elucidation of his personal problems rather than a prescription for a bottle of medicine.

It is a curious sidelight on modern civilization that so large a fraction of the community should exhibit these emotional difficulties ; for the symptoms of the functional nervous disorders are the physical expression of emotional conflicts, and these centre round the demands for love, protection and power.[1]

The first problem is to recognize the functional nervous disorder when it presents itself for diagnosis in the consulting-room. This can sometimes be extremely easy and sometimes extraordinarily difficult. In my experience, when the diagnosis is easy the treatment is often very difficult ; on the other hand, when the diagnosis has taken an hour there is often no treatment necessary, as the patient is cured.

Another paradox: when the patient attributes his illness to some disorder of one of his organs I at once ask myself: "Can this patient be suffering from a functional nervous disorder?" Whereas if she humbly apologizes: "It is only my nerves, doctor," I am more inclined to keep my eyes open for a masked hyperthyroidism, a latent hypoglycæmia, or a toxæmia from a hidden septic focus.

Always I distrust the patient's diagnosis, for to the patient suffering from a functional disorder nothing is less acceptable than "nerves." "I am not that sort of person, doctor." Often enough, in fact much too often, the patient will support his opinion by saying that Dr. X. has told him that his heart is "weak" or "flabby," or she suffers from "colitis," and to that blessed label of organic disease the patient clings like the drowning man to his proverbial straw.

But it must be remembered that the patient is unaware of the true cause for this visceral expression of emotion or "organ-jargon," as Dr. Crookshank has called it.[2] The organs of the body are a keyboard on which the emotions of the sufferer play, sometimes a single note, sometimes an insistent chord, and sometimes just jazz.

We cannot say why the lachrymal glands should secrete tears when we feel sad, but we do not dignify the process of weeping by calling it an acute dacryoadenitis or recommend extirpation of the lachrymal glands as a cure for depression. But not every organ in the body enjoys this immunity. What of the colon that expresses its owner's fear? It has been labelled "Colitis," purged, lavaged, short-circuited or even resected. What of the uterus that expresses unsatisfied desire with a menorrhagia; "endometritis" and the insult of the curette; or, worse still, "fibrosis" and a hysterectomy? What of the kidney whose vagrancy indicates its owner's inability to cope with hopeless environment? "Ptosis," we cry, and away with it for a nephropexy. After all, if the worst comes to the worst, the patient can spare a kidney, can exist without a uterus, can even carry on without a colon; but when the emotions find outlet in organs that are indispensable our surgical zeal must relent, and the doctor must lengthen his face, shake his head and regret that there is "nothing organic."

D.A.H., or disordered action of the heart, was an example of a masterly compromise; nothing organic, but the patient was palpitating with fear. But what about D.A.L., or disordered action of the lungs—the psychogenic asthma? Or D.A.B., disordered action the bladder—enuresis? Or D.A.E., disordered action of the eyes—otherwise miner's nystagmus? Or another D.A.L., disordered action of the labyrinth—dignified by the eponym, Ménière's syndrome? All these disorders are frequently associated with emotional disturbances, and may have no organic basis.

And so on listening to the patient's tale of woe I always ask myself: "Can these be the expressions of some emotion of which he is unaware?" These patients are not malingerers; they are unconscious of any relation between their symptoms and the emotional conflicts which may be going on in their minds.

Investigation of these nervous disorders must proceed on the time-honoured lines of the detective novel; we must first of all search for motives, and then proceed to establish relations of time according to the best precedents.

We may take for granted that the patient confronted with a "spot" diagnosis of motive, however accurate, will deny it stoutly. It is useless to impose the correct answer on the patient: it will be resisted. Cure depends on his working out his own solution, he must *feel* that it is correct, and so we must lead him gently up the garden-path.

Here are some of the stock solutions Not every case will fall into the categories I am going to describe, but a large number will be found under the following invoice. I am going to leave out of consideration the infant and the child, as they would require a lecture to themselves, and I will deal first of all with the adolescent.

Taking then the female of the species. The young girl confronted suddenly with developing contours and the onset of menstruation dimly apprehends the underlying problem of sex, and, if unprepared, is sometimes apt to evade the issue by embarking on a strict diet in the vain hope of arresting the progress of nature. And we then meet the patient so well illustrated by Professor Langdon Brown[3] as suffering from anorexia nervosa, the label for the involuntary hunger strike.

But puberty is not the only time that anorexia nervosa is prevalent. It is not uncommon in women of maturer years, and in practically every case inquiry will establish that the sufferer is faced with an apparently inexorable fate from which she has little hope of escape and no avenue for expressing her despair. Her emotions select this unconscious hunger strike as an avenue of expression.

To return, however, to the problems of adolescence. If the male has no reason to be concerned about the alterations in his bodily configuration, yet there are disturbing changes in his physiology which can prove most upsetting. I refer, of course, to the problem of masturbation. The normal adolescent goes through this phase without much bother or self-reproach, but a number of youths develop an anxiety state about this matter. They can sometimes be recognized by their preoccupation with physical culture. They go in for strenuous athletics, not because they enjoy them, but "to work off their energy." Sometimes they consult the family doctor about the best method of physical training, and go in for Sandow's or My System. They make all kinds of fanciful alterations in their diet to avoid stimulation, and follow an ascetic régime of cold baths and long walks, but all to no purpose. The occasions for remorse and self-reproach still recur. Many of these cases are best tackled frankly on the subject. Most of them live in constant dread of remote but certain consequences of spinal degeneration, of insanity, of loss of virility. A short talk, refuting the bogy tales of the schoolmasters and clergymen, clears up many of their anxieties, and for many this is sufficient, but in others further treatment is required. Masturbation in many of these cases is no more than an urge to bring a little excitement into an otherwise drab and colourless existence; most of the patients are clerks chained to an office desk, not earning enough salary to be entirely independent of the paternal roof or to afford the pleasures of female society, and subject to the restrictions of a strictly regulated, often Nonconformist household. Culpin[4] goes so far as to say that the treatment of masturbation resolves itself into the treatment of the emotions of adults in regard to it. It has been pointed out that masturbation is the eroticism of the lonely, unhappy, solitary, fearful, the selfishly self-centred, the diffident and the distrustful.[5] It is a symptom, not a disease, a symptom of cowardice about life in general and sexual life in particular. It is useless to reproach the victim for his lack of self-control—he has already done that himself *ad nauseam.*

The next fence in the Grand National, so to speak, is that of approaching marriage. Here again a great many riders clear the obstacle in their stride, but a certain number display a tendency either to refuse the jump or make a bad landing.

The modern woman has more grounds for indecision than her grandmother. Released from the necessity of living at home, in many cases financially independent and better educated, she needs more assurance that the advantages of marriage outweigh the relative disadvantages of loss of independence and varied domestic responsibilities. Moreover, the modern woman is already disillusioned in advance about the physical side of marriage, and the tendency nowadays appears to be to expect the worst rather than to hope for the best. Here comes the main difficulty about the preparation for marriage movement, for at present it deals in a scientific objective manner with the facts but omits the description of the emotional tensions involved. Havelock Ellis[6] points out that the mere knowledge of the facts is not enough. This I can endorse, as I well remember the case of a young woman who had been most carefully instructed by an up-to-date mother with regards to the "facts of life," but when first faced with the realities of the emotional situation was so emotionally unprepared that she broke down completely and became so dissociated that she had the feeling that "her body did not exist."

Amongst the functional disorders that I have recognized in young women engaged to be married have been a functional leucorrhœa, a lingual neuralgia, a cardiospasm, a "colitis," anorexia with cough, and to these I would add a rheumatoid arthritis that improved when the girl's fiancé, who was a rotter, broke off the engagement. Prospect of marriage seems less formidable to the male. I have notes of one case of severe and frequent migraine which, in the light of subsequent events, appeared to have been due to worry over premarital intercourse. Another man suffered from migraine associated with fears of insanity and various fatal illnesses. He really couldn't afford to marry, but had rashly fixed up the date.

The next fence, and a stiffer one, is married life itself. Here a good many riders crash who have taken the other obstacles like a bird, while those who have already put a foot wrong very often come to grief badly.

Adler has defined marriage as a "constructive task for two persons who are determined to live together in order to relieve and enrich each other's lives."[7] But Wexberg states that "of marriage, as comradeship, the neurotic has no comprehension."[8] If one partner cannot live up to this standard of sexual and social co-operation, he or she must safeguard himself or herself by a "series of evasions, arrangements, compromises, hesitations, fraudulent devices, bogus insurances and confidence tricks which they put across themselves."[9] And so again we have the flight into illness.

Usually this flight into illness does not come on gradually, but develops after some genuine occasion. An operation for a real appendicitis, the birth of a child, a pneumonia, provides a useful peg. Convalescence is prolonged, and the woman states that

"she has never been the same since her operation or her baby." A state of chronic invalidism develops which resists all tonics, recurs after every change of air, and is the despair of the family doctor.

The blame which the patient refers to the appendicitis or the "shock" of labour is really a displacement of affect. The woman is really blaming the circumstances of married life. She may really be resenting clumsy technique or cooling of passion of her partner. She may feel insecure if her husband devotes himself to his business or spends the week-ends away from home. More often the conflict is deeper and more unformulated. It is based on the conflict between the will to power which appears in every human relationship and the submission and dependence which are the biological requirements of sexual life in women.[10] She may have plenty of [reasons, good, bad and indifferent, but the point at issue is that she is failing to face the situation, is thrusting it at the back of her mind, and solving the burning question in a useless, painful and expensive manner. And why do I specify women? Because the man has more opportunities of blowing off steam. If things have gone badly he can take it out of the typist or the chauffeur or the office-boy or knock a ball round the golf course. But the woman, tied to a house, at close quarters with howling infants and incompetent maids, dare not let herself go.

The next fence proves a regular Becher's Brook to most of the compulsory competitors. The casualties are very numerous. The menopause marks a definite stage in the life history of the patient, and it is inevitable that a certain amount of stock-taking, conscious or unconscious, takes place at this period. Many of the symptoms of the climacteric are really evidence of a considerable emotional conflict which may be going on below the level of consciousness. A review of the past may bring regrets for missed opportunities, serious errors of judgment, loss of objectives; and a forecast into the future may bring to light fears of loss of health, of security and of earning capacity.

Many patients report at this time strange pains about the heart, the abdomen and the pelvis which cannot be explained on anatomical grounds. In nearly every case these patients have an underlying fear of dropping down dead, of gastric ulcer or cancer of the uterus. A thorough examination and a *specific* assurance that there is no evidence of disease in the organ complained of generally cures these pains. But some of these patients, when reassured about the organ, return next week with pain in a new site. Here fear is being projected on to an organ of the body. Really the fear of organic disease is a rationalization of the conscious mind. Underlying this is an unformulated fear of the future, of poverty, of neglect, of failing charms, of a future meaningless without objectives. These are generally women who have devoted themselves to their homes, their children, their husbands; never having allowed themselves leisure to cultivate any interest outside their homes, they now see their children growing up and about to leave them, their husbands absorbed in business interests, and the future a blank.

Another rather common situation is that of the unmarried daughter who has stayed at home to look after her parents. On the death of her parents, often, in the nature of things, about the time of her climacteric, her little world comes tumbling about her ears.

The evidence for a climacteric in the male has been much disputed, but I do support Kenneth Walker's contention that it occurs.[11] With a certain type of man, the failure of sexual powers is a great blow to their self-esteem, and I have had half a dozen patients who have suffered from various nervous symptoms which ultimately appeared to have been based on this experience. One of them expressed himself: "I feel I must have gone through what women call the change of life." I believe it may be a factor in the nervous invalidism that sometimes follows prostatectomies in younger men. A suddenly enforced abstinence at this time may also provoke symptoms.

And so we come to the last and final obstacle, the problem of old age and a dignified senescence. The woman generally makes a better show of it than the man. It was not for nothing that Miss Sackville-West chose a heroine, not a hero, for her study of old age, *All Passion Spent.* If the woman has survived the obstacles of adolescence, menstruation, marriage, pregnancies and the menopause, old age will present few difficulties.

The two fears associated with old age are loss of security from a failure of earning capacity and the fear of dying. This latter is more a terror of the physical process of dying than a fear of death in the abstract.

For the man old age has its own problems. Chief of these is the difficulty of adjusting to a life of leisure. Voluntary retirement or compulsory pensioning often proves disappointing. For many years the old man has looked forward to the time when he can enjoy his leisure, but in so many cases when the opportunity arrives he is unprepared to make use of it.

Many men of sixty-five to seventy suffer from functional nervous disorders for the first time in their lives. The illness is often precipitated by the news of the death of a relative or old friend. Headaches and giddiness are interpreted as the onset of a stroke, loss of memory as softening of the brain, palpitation as heart disease, spots in front of the eyes as cataract. But behind these fears of disease lurks the fear for the future, of dying itself.

The average man, when he thinks of dying, visualizes some dramatic death-struggle seen on the stage or screen, or some harrowing exitus described in some novel. For it must be remembered that the average civilian rarely sees a death. I have been surprised sometimes when elderly relatives have told me that this was the first death-bed they had witnessed. Such people in their ignorance often invest the final passage in their imaginations with fearful physical agony.

Of course, in truth, death in the sixth and seventh decades steals gently on his victims. There is a merciful clouding of consciousness, and the majority of elderly patients that I have watched over the border have been in a coma for many hours. Curiously enough, it is the religious man who is often most appalled at the idea of dying; perhaps the cynic would say that his religion was in the nature of an insurance. I have never seen the so-called *spes phthisica* in an elderly patient, though it is the common reaction of the younger patient, not only in tuberculosis but in other diseases.[12] The *spes phthisica* has no particular relation to tuberculosis. It is merely a stock evasion of the approach of death by a person who refuses to accept the situation.

You will realize that, with Miss Rose Macaulay, I have come to the conclusion that every age has its potentialities as a "dangerous age." In dealing with all these patients we must remember what a former Long Fox lecturer has told us: "It is not an illness we treat, but an individual who is ill."[13] Our object must be first to recognize the nature of their disorder, then, by establishing good relations with our patient, to win his confidence. With encouragement and persuasion that confidence can be enlarged and transferred to the difficult situation he is refusing to face.

A recent writer[14] has pointed out that much of a family doctor's work is a "specialized kind of friendship." Goodwill, informed by science and tempered by judgment, must be the therapeutic agent in the hands of the general practitioner who deals with the neurotic.

### REFERENCES.

[1] Graham Howe, *Motives and Mechanisms of the Mind*, London, 1931, p. 34.

[2] Crookshank, *Individual Psychology Publications*, No. 9, London, 1933.

[3] Langdon Brown, *Individual Psychology Publications*, No. 2, London, 1931.

[4] Culpin, *Recent Advances in the Study of the Psychoneuroses*, London, 1931, p. 76.

[5] Crookshank, *Individual Psychology Publications*, No. 3, Jan., 1932, p. 45.

[6] Havelock Ellis, *More Essays in Love and Virtue*, London, 1931, p. 10.

[7] Adler, *Problems of Neurosis* (trans.), London, 1929, p. 23.

[8] Wexberg, *Individual Psychological Treatment*, London, 1929, p. 78.

[9] Crookshank, Preface to *Problems of Neurosis*, London, 1929, p. xxvi.

[10] Van der Velde, *Sex Hostility in Marriage*, London, 1931, p. 65.

[11] Kenneth Walker, *Male Disorders of Sex*, London, 1930, p. 20.

[12] Banister, *British Association Ninety-Eighth Report*, Bristol, 1930.

[13] Devine, *Recent Advances in Psychiatry*, London, 1929, p. 14.

[14] Winnicott, *Clinical Notes on Disorders of Childhood*, London, 1931.

